# Frozen Feet in the Army

**Published:** 1918-02

**Authors:** 


### TRENCH FEET

***Frozen Feet in the Army.*** By M. Témoin, Correspondent National. Trans. and Abs. from the *Bulletin de l'Académie de Médecine*, t. LXXIII, 3e série, p. 155 (Séance of Feb. 2, 1915).

T. expressed himself as convinced, after examining a large number of cases of men reported as having frozen feet, that cold was not the principal cause of their malady, but rather the arrest of the circulation in the lower extremities caused by the tightness of the shoes and gaiters. The lesions resulting were essentially like those produced by any appliance that hinders the circulation.

If so many soldiers during a mild winter had cases of frozen feet, T. wondered how a single soldier had escaped during the war of 1870, when the temperature fell to 15° or 20° below 0 C. He was also surprised to note that although in 1870 freezing of the hands, ears, and nose had been frequent, not a single case of the kind had come to his notice during the present war. It was well known that for cold to produce the vaso-constriction necessary to cause gangrene, the human tissues must be immobilized at a temperature of from 18° to 20° degrees below freezing. None of the cases of trench feet observed had been subjected to a temperature below freezing, but had been in water or mud. Fisherman often may remain standing in water whole nights during severe winter weather without having frozen feet.

Cases of frozen feet in 1870 usually occurred when soldiers were obliged to sleep in the snow. In the trenches complete immobilization in the cold was never necessary, and even slight movement was sufficient to stimulate circulation. The typical history of a case of trench feet was as follows: after a long spell in trenches which had been converted into bogs or rivers, the men would at first feel an unpleasant but not painful sensation of swelling, then a marked sense of tightness caused by the shrinkage of the shoes and gaiters. Upon removing the shoes, the patient would find that some of his toes, or even large parts of his foot had swollen and turned dark, and that they felt numb. Sometimes large black or violet blotches, as in gangrene, would be seen; and frequently blisters, as in burns. When cases appeared at the hospital, these symptoms were generally present. The toes, completely black, would often appear separated from the foot by a suppurating line of demarcation. In many cases blotches of gangrene would appear scattered over the surface of the foot.

T. could not believe that these lesions were due to cold, but he was convinced that they were the result of tight wrappings about the feet and limbs, and that the cold was merely contributory. Whether the effect is produced by cold or compression, the results are practically the same. In some cases the stoppage of circulation is merely superficial, and complete healing may be expected. In others, however, deeper vessels are involved, whereby parts of the foot are completely devitalized and subsequently eliminated.

T. then described in detail the interference with the circulation due to tight lacings, puttees, etc., noting especially that the men in order to keep the water out of their shoes, purposely drew the lacings and puttees as tight as possible. In spite of this, the water penetrated, causing additional swelling, and, by shrinking the cloth and leather, completed the interruption of the circulation.

It was often noted that one foot would be completely free from lesions, when the other was badly affected. Such occurrences could hardly be explained by a theory that cold was the cause, whereas it would be most natural for the covering of one foot to be enough tighter to make all the difference between good and bad circulation.

Furthermore, the effects were gradually produced. The first symptoms usually appeared after about 3 days of exposure, but these were only superficial and easily remedied. On the 4th day there appeared a general devitalization of the soft parts. Gangrene of the toes and feet was rarely noticed until after 6 days of exposure in water without change of shoes.

T. had noticed an instance in which one of two companions, who had been exposed in exactly the same way, had almost complete gangrene of the feet, whereas the other had only slight lesions. He learned that the latter had frequently removed his shoes, and so had reestablished the circulation. In another case it was noticed that the gangrene extended above the ankles to the exact line where the first fold of the puttee was made.

The most serious feature of these cases is the slowness and painlessness with which the malady is produced. The patient usually does not notice it until it is fully established. One patient, who was later found to have gangrene of all the toes, walked 12 kilometers.

T. recommended the following measures of prevention :

1. Greasing the foot to prevent saturation of the tissues.

2. Diminishing the length of shifts in the trenches to 48 hours if possible.

3. Providing complete freedom for circulation. If men must stand in water, they should not tighten their lacings or puttees. It would be better to let the water enter freely into the shoes, or to wear no shoes at all. At least, the shoes should frequently be removed.

T. remarked that M. Baratte had succeeded in suppressing this malady by having all the shoes dipped in warm grease and by having the men ordered to unlace their shoes for a quarter of an hour twice every day.

The Academy, acting upon the advice of a committee appointed to examine M. Témoin's recommendations, voted unanimously to transmit to the minister of war a mémoire to the effect that cold was only a secondary factor in causing trench feet.

